# Modeling recovery of natural gas from hydrate reservoirs with carbon dioxide sequestration: Validation with Iġnik Sikumi field data

**DOI:** 10.1038/s41598-019-55476-1

**Published:** 2019-12-11

**Authors:** Avinash V. Palodkar, Amiya K. Jana

**Affiliations:** Energy and Process Engineering Laboratory, Department of Chemical Engineering, Indian Institute of Technology, Kharagpur, 721302 India

**Keywords:** Pollution remediation, Carbon capture and storage

## Abstract

Fundamental understanding of guest gas replacement in hydrate reservoirs is crucial for the enhanced recovery of natural gas and carbon dioxide (CO_2_) sequestration. To gain physical insight into this exchange process, this work aims at developing and validating a clathrate hydrate model for gas replacement. Most of the practical concerns associated with naturally occurring gas hydrates, including hydrate formation and dissociation in interstitial pore space between distributed sand particles in the presence of salt ions and in irregular nanometer-sized pores of those particles, irregularity in size of particles and shape of their pores, interphase dynamics during hydrate formation and decay, and effect of surface tension, are addressed. An online parameter identification technique is devised for automatic tuning of model parameters in the field. This model is employed to predict the laboratory-scale data for methane hydrate formation and decomposition. Subsequently, the model is validated with the field data of the Prudhoe Bay Unit on the Alaska North Slope during 2011 and 2012. In this Iġnik Sikumi field experiment, mixed CO_2_ (i.e., CO_2_ + N_2_) is used as a replacement agent for natural gas recovery. It is observed that the proposed formulation secures a promising performance with a maximum absolute average relative deviation (AARD) of about 2.83% for CH_4_, which is even lower, 0.84% for CO_2_ and 1.67% for N_2_.

## Introduction

Natural gas hydrates are crystalline solid compounds that represent a vast natural gas resource found in submarine sediments and permafrost layers. It is reported that about 3 × 10^13^ m^3^ of natural gas is remained in recoverable form globally, which is close to the amount of shale-gas (~665 TCF) locked in the United States^1,2^. To recover this huge amount of next generation fuel, there are several existing techniques, such as thermal treatment, depressurization and inhibitor injection^3–5^. Interestingly, all these methods involve CH_4_ decomposition by external stimulation that can trigger catastrophic slope failure. Importantly, uncontrolled hydrate decomposition may further accelerate the greenhouse effect through the transfer of released methane gas to the atmosphere.

In this light, the guest gas swapping can play a promising role for natural gas extraction^6^. Using CO_2_ as a replacement agent, it is feasible to recover CH_4_ by simultaneously sequestering CO_2_, which is a potential greenhouse gas^7,8^. By this way, the gas hydrates can serve as geological reservoir for CO_2_ sequestration and remain at stable state as non-destructive structures that may additionally lead to avoid the seafloor instability problem, among others.

The feasibility of this replacement technique is verified in laboratory scale. Ota *et al*.^9^ have studied the CH_4_ replacement with high pressure CO_2_ and shown that the replacement rates depend on pressure and phase conditions with the driving force being strongly related to fugacity differences of the two guest components between fluid and hydrate phases. Zhou *et al*.^10^ have used the same replacement agent at its different concentrations in carbon dioxide-in-water emulsions and determined the appropriate exchange condition. Subsequently, Yuan *et al*.^11^ have performed experiment on CH_4_-CO_2_ exchange with 3.35 wt% Na_2_SO_4_ aqueous brine and quartz sands. Before them, Lee *et al*.^12^ have performed experiment for the replacement mechanism of CH_4_ hydrates with CO_2_ and found by using the spectroscopic method that CO_2_ molecules mainly occupy the large cages, and thus they replace CH_4_ in those large cages, keeping CH_4_ molecules in small cages almost intact. Adding N_2_ to CO_2_ leads to increase the replacement efficiency further, providing a considerably wider safety net for the stability of CO_2_ in the clathrate phase^13^. This is because, like CH_4_, N_2_ molecules prefer small cages, and thus CH_4_ and N_2_ compete in those small cages, thereby an enhanced recovery of natural gas. In this direction, the effect of mixed CO_2_ (CO_2_ + N_2_) on CH_4_ replacement is investigated subsequently^2,12^. It is reported that the CO_2_-alone has led to 64% CH_4_ recovery yield, whereas the CO_2_ + N_2_ gas mixture has achieved 84%^3^. Further, it is investigated that there is an optimum reservoir pressure (at a certain temperature) that leads to maximize the CO_2_ capture and storage from injected flue gas or CO_2_-N_2_ mixture in the reservoir^14^.

It is fairly true that the gas hydrates occur at harsh engineering conditions and most of their sites are not easily accessible. Despite the engineering barriers, first onshore field test was performed in 2012 on the Alaska North Slope in the US and the potential of gas exchange mechanism is evaluated^15,16^. Subsequently, on the Eastern Nankai Trough in Japan, the first offshore gas hydrate field test was conducted in 2013 by the use of depressurization technique^17^. In order to understand the real-time gas swapping technique better, a laboratory experiment is performed at conditions experienced during the onshore field trial in Alaska^18^. Their experiment has isolated the kinetic guest molecule exchange process from additional hydrate formation and mechanical changes to the hydrate bearing sand.

There are a very few models reported in literature on hydrate formation (e.g.^19–22^) and decomposition (e.g.^23,24^). As far as theoretical formulation of gas exchange is concerned, a little attention has been paid so far. In this regard, Yuan *et al*.^11^ have proposed a model for CH_4_-CO_2_ swapping with porous medium. Subsequently, a simulator (STOMP-HYDT-KE) is developed^18^ using a combination of production technologies, namely depressurization, thermal stimulation, inhibitor injection, and guest molecule exchange. The simulator is used for CH_4_/CO_2_/N_2_ guest molecule exchange replacement in the presence of pure water^16,18^. Recently developed gas exchange models^11,25^ need to be verified with the real-time data sets.

The abovementioned models do not consider the salt affect. More importantly, there are various practical issues that need to be addressed in replacement modeling. They include: hydrate nesting in nanometer-sized pores of the distributed porous particles along with in interstitial pore space between them, irregularity in size and shape of the pores, interphase dynamics during hydrate formation and decay, and effect of surface tension. Furthermore, it is wise to consider chemical potential difference as an effective thermal driving force since it takes into account the effect of temperature, pressure and phase composition together. In this contribution, all these issues are taken into account in developing the exchange model to replace the natural gas by pure CO_2_, and mixed CO_2_ and N_2_ gas. To show the versatility of this formulation that considers both the thermodynamic and kinetic aspects, the Iġnik Sikumi field data (Alaska North Slope) are used. The proposed generalized model provides physical insight into the gas replacement process and shows a promising performance in predicting the gas replacement mechanism. Because of its online parameter tuning ability, this model is recommended to use in the gas hydrate field.

## Motivation and Objective

**Figure 1:**
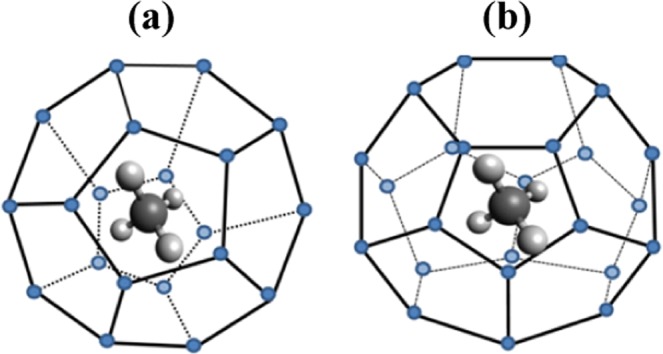
Schematic of the single (**a**) small and (**b**) large cavity of methane gas hydrate.

Naturally occurring hydrates form once an appropriate guest gas like methane comes in contact with the water molecules at a reasonably high pressure (i.e., more than the atmospheric pressure), and/or the low temperature^26^. The most common guest gases such as methane and carbon dioxide are recognized as s-I hydrate formers^5^, and their unit cell comprises of two small and six large cavities made from the hydrogen-bonded water molecules network, as depicted in Fig. 1. The natural gas hydrates (NGH) are truly a great source of hydrocarbon energy. The potential energy content is about twice that of all the other fossil fuels combined together^1^. Deep sea-beds and permanently frozen grounds are the favourite and habitable hosting sites for the NGH. In these reservoirs, usually the hydrates are enclosed in the unconsolidated porous sand layers/sediments, and accompanied by the saline water. Among these, former element (porous medium) acts in favour of the hydrate formation, and later one (seawater) typically hinders their growth. Thus, it is inevitable to overlook their influence on the naturally occurring gas hydrates.

The gas hydrates prefer to cultivate themselves in between the interstitial pore spaces as well as in the internal nano spaces/pores of the porous materials. Naturally, the guest gas and water saturations, interfacial tensions and contact angle between the hydrate and liquid phase, size and shape of the irregular internal pores, etc. stand as the authoritative elements. Besides, the salt ions, a key constituent of the saline water, interfere in the formulation of the strong hydrogen-bonded water molecules network that leads to form the hydrate cavities. Albeit they do not participate in the phase transformation, their presence usually reduces the rate of gas hydrate formation and growth. Taking all these premises into account, here we formulate the theoretical framework for the formation and decomposition of gas hydrates, and guest gas exchange in those hydrates, involving the natural gas extraction with CO_2_ sequestration. As mentioned and shown below, the proposed formulation has blended both the thermodynamic and kinetic aspects.

## Results

### Formation and decomposition

The governing equation for the gas hydrate dynamics is proposed as,

1 $$\frac{d{n}_{\text{gg},{\rm{H}}}}{dt}=\{\begin{array}{cc}+k\,{A}_{{\rm{e}}}\,\Delta \mu \,{n}_{{{\rm{H}}}_{2}O,{\rm{L}}} & {\rm{Formation}}\,\& \,{\rm{growth}}\\ -k\,{A}_{{\rm{e}}}\,\Delta \mu \,{n}_{{{\rm{H}}}_{2}O,{\rm{H}}} & {\rm{Decomposition}}\end{array}$$

in which, *n*_gg,H_ is the molar composition of the guest gas (suffix ‘gg’) in hydrate phase.

Here, the intrinsic rate constant, *k* is adopted^22^ as a function of temperature (*T*) and activation energy (Δ*E*_a_): *k* = *k*_0_ exp(−Δ*E*_a_/*RT*).

An important issue is the renewal of reaction surface during the gas hydrate growth and decay. It is taken into account through the time-dependent effective surface area (*A*_e_) of an unconsolidated porous material as,

2 $${A}_{{\rm{e}}}={\beta }_{0}\,\exp \,(Ct)A$$

in which, *C* is a tuning parameter (constant) that is negative for hydrate growth and positive for decay, and *β*_0_ the surface area adjustment factor. This surface area of the unconsolidated irregular porous sediment (*A*) is evaluated as: $A=e\,{V}_{{\rm{Tp}}}^{(2/3)}$, in which, the scaling factor (*e*) is 6.2918^27^ and the total volume of the porous material (*V*_Tp_) is a function of the porosity (*ϕ*) and void volume of the bed (*V*_void_) as: *V*_Tp_ = *V*_void_/*ϕ*.

Next issue is the driving force (Δ*μ*) of the gas hydrate formation, growth and decomposition. It is considered as,

3 $$\Delta \mu =\langle \begin{array}{cc}\frac{{\mu }_{{\rm{w}}}^{{\rm{H}}}}{RT}-\frac{{\mu }_{{\rm{w}}}^{{\rm{L}}}}{RT} & {\rm{Formation}}\,\& \,{\rm{growth}}\\ \frac{{\mu }_{{\rm{w}}}^{{\rm{L}}}}{RT}-\frac{{\mu }_{{\rm{w}}}^{{\rm{H}}}}{RT} & {\rm{Decomposition}}\end{array}$$

where, ${\mu }_{{\rm{w}}}^{{\rm{H}}}$ and ${\mu }_{{\rm{w}}}^{{\rm{L}}}$ are the chemical potentials of water in the filled hydrate phase and the liquid phase, respectively, which are evaluated from,

4 $${\mu }_{{\rm{w}}}^{{\rm{H}}}={\mu }_{{\rm{w}}}^{0}+RT[\mathop{\sum }\limits_{i=1}^{2}{v}_{i}\,\mathrm{ln}\,(1-\mathop{\sum }\limits_{j=1}^{{N}_{{\rm{c}}}}{\theta }_{ij})]$$

5 $${\mu }_{{\rm{w}}}^{{\rm{L}}}={\mu }_{{\rm{w}}}^{0}-RT\{(\frac{\Delta {\mu }_{{\rm{w}}}^{0}({T}_{0},0)}{R{T}_{0}})-({\int }_{{T}_{0}}^{T}\frac{\Delta {h}_{{\rm{w}}}^{{\rm{L}}}(T)}{R{T}^{2}}\,dT)+({\int }_{0}^{P}\frac{\Delta {V}_{{\rm{w}}}^{{\rm{L}}}}{R{T}^{2}}dP)-(\mathrm{ln}\,[\begin{array}{c}({\gamma }_{{\rm{w}}}{x}_{{\rm{w}}})+\exp (-\frac{2k{V}_{{\rm{w}}}{\sigma }^{\infty }\,{r}_{{\rm{core}}}^{2-{D}_{{\rm{f}}}}}{RT({r}_{{\rm{core}}}^{2-{D}_{{\rm{f}}}}+2k\delta )})\\ \,\,\,+\exp (-\frac{{\rm{MW}}}{1000}(\sum _{l}{m}_{l})\phi )\end{array}])\}\,$$

The fractional occupancy (*θ*) of each guest gas forming s-I type hydrate structure is a strong function of Langmuir constant (*LC*) and the fugacity of the guest gas (*f*_*j*_). Among the terms in Eq. (5), the reference temperature (*T*_0_) and the standard chemical potential of water (${\mu }_{{\rm{w}}}^{0}$) are fixed^28,29^, and the enthalpy ($\Delta {h}_{{\rm{w}}}^{{\rm{L}}}$) and molar volume ($\Delta {V}_{{\rm{w}}}^{{\rm{L}}}$) difference between the empty hydrate lattice and liquid water, and the activity of water (*a*_w_) (last component of Eq. (5)) are the function of operating temperature and well pressure^25^. Further, *γ*_w_ and *x*_w_ indicate the activity coefficient and concentration of water, respectively.

As far as the second term of last component in Eq. (5) is concerned (porous media contribution), it accounts for the gas hydrate formation in the nanometer sized internal and interstitial pores present in natural porous material (i.e., silica sand), and the associated influence of the surface tension. This term comprises of the radius of a hydrate core present in the internal pores (*r*_core_), the interfacial energy between the planar interfaces (σ^∞^), the thickness of an interfacial region between the solid and liquid phase (*δ*) and a linear function of pore radius (*k*) as: *k* = *a*+*br*_pore_. Further, the uniform irregularities in the shape of the internal pores are considered through the fractal theory^28^ with *D*_f_ as a fractal dimension^28^.

The third term of last component in Eq. (5) accounts for the crucial element of the marine hydrate reservoir (i.e., seawater). In which, MW denotes the molecular weight of water, *m* the concentration of solute species *l* (i.e., cation, anion or neutral) and *ϕ* the osmotic coefficient, which is evaluated from the Pitzer model^30^.

Naturally, the total moles of water, ${n}_{{{\rm{H}}}_{2}{\rm{O}},{\rm{T}}}$ is distributed in liquid (${n}_{{{\rm{H}}}_{2}{\rm{O}},{\rm{L}}}$) and hydrate phase (${n}_{{{\rm{H}}}_{2}{\rm{O}},{\rm{H}}}$). Therefore, these are interrelated quantities, and by knowing any two of them, one can find the third one. For this, we use the following expression as,

6 $${n}_{{{\rm{H}}}_{2}{\rm{O}},{\rm{T}}}=\langle \begin{array}{c}{n}_{{{\rm{H}}}_{2}{\rm{O}},{\rm{L}}}+{n}_{{{\rm{H}}}_{2}{\rm{O}},{\rm{H}}}\\ {n}_{{{\rm{H}}}_{2}{\rm{O}},{\rm{L}}}+{n}_{{\rm{H}}}\,{n}_{\text{gg},{\rm{H}}}\end{array}$$

Solving the differential Eq. (1), we get the respective expression for gas hydrate formation and dissociation as,

7 $${n}_{\text{gg},{\rm{H}}}=\frac{\alpha \,{n}_{{{\rm{H}}}_{2}{\rm{O}},{\rm{T}}}}{{n}_{{\rm{H}}}}\{1-\exp \,[-\frac{{n}_{{\rm{H}}}{k}_{0}{\beta }_{0}}{CRT}\,A\,\exp (\frac{-\Delta {E}_{{\rm{a}}}}{RT})\,({\mu }_{{\rm{w}}}^{{\rm{H}}}-{\mu }_{{\rm{w}}}^{{\rm{L}}})\,(1-\exp \,(-Ct))]\}$$

8 $${n}_{\text{gg},{\rm{H}}}={n}^{0}\,\exp \,\{\,(\frac{{n}_{{\rm{H}}}{k}_{0}{\beta }_{0}}{\alpha \,RT\,C})\,A\,\exp \,(\frac{-\Delta {E}_{{\rm{a}}}}{RT})\,({\mu }_{{\rm{w}}}^{{\rm{L}}}-{\mu }_{{\rm{w}}}^{{\rm{H}}})\,(1-\exp \,(+Ct))\}$$

In which, *α* is an adjustable parameter and it is defined as the ratio of the highest value of the net amount of guest gas exhausted during the hydrate formation and the total amount of that gas ideally locked in all cages.

### Gas exchange. Fundamental mechanism

The difference, in terms of: (i) heat of formation of the CO_2_ hydrate and heat of dissociation of the CH_4_ hydrate, and (ii) operating pressure and temperature of the formation of CO_2_ and CH_4_ hydrates, drives the CH_4_-CO_2_ (pure/mixed) gas exchange process for the natural gas recovery. This leads to perform double duty i.e., extracting the energy in the form of natural gas and sequestrating the major greenhouse gas contributor (i.e., CO_2_) in the NGH reservoir. Basically, the pure CO_2_ or mixture of the CO_2_-N_2_/H_2_ is injected in the existing natural gas hydrate bearing sediments to disturb their equilibrium. Once that gets derailed, the CH_4_ hydrate decomposes to the free CH_4_ gas and water. At that time, the CO_2_ present in the gas phase makes use of these free water molecules to form the CO_2_ hydrates^6,9,11^. Thanks to the memory effect of the dissociated water, owing to which, the CO_2_ experiences relatively small resistance to form hydrate cages^6,9,11,31^. However, monopoly of the CO_2_ (pure/mixed) in forming the hydrates continues until the CH_4_ in the gas phase reaches the critical methane concentration (CMC). Afterward, the CH_4_ again participates in the hydrate formation^6,9,11,32^.

### Modeling

**Figure 2:**
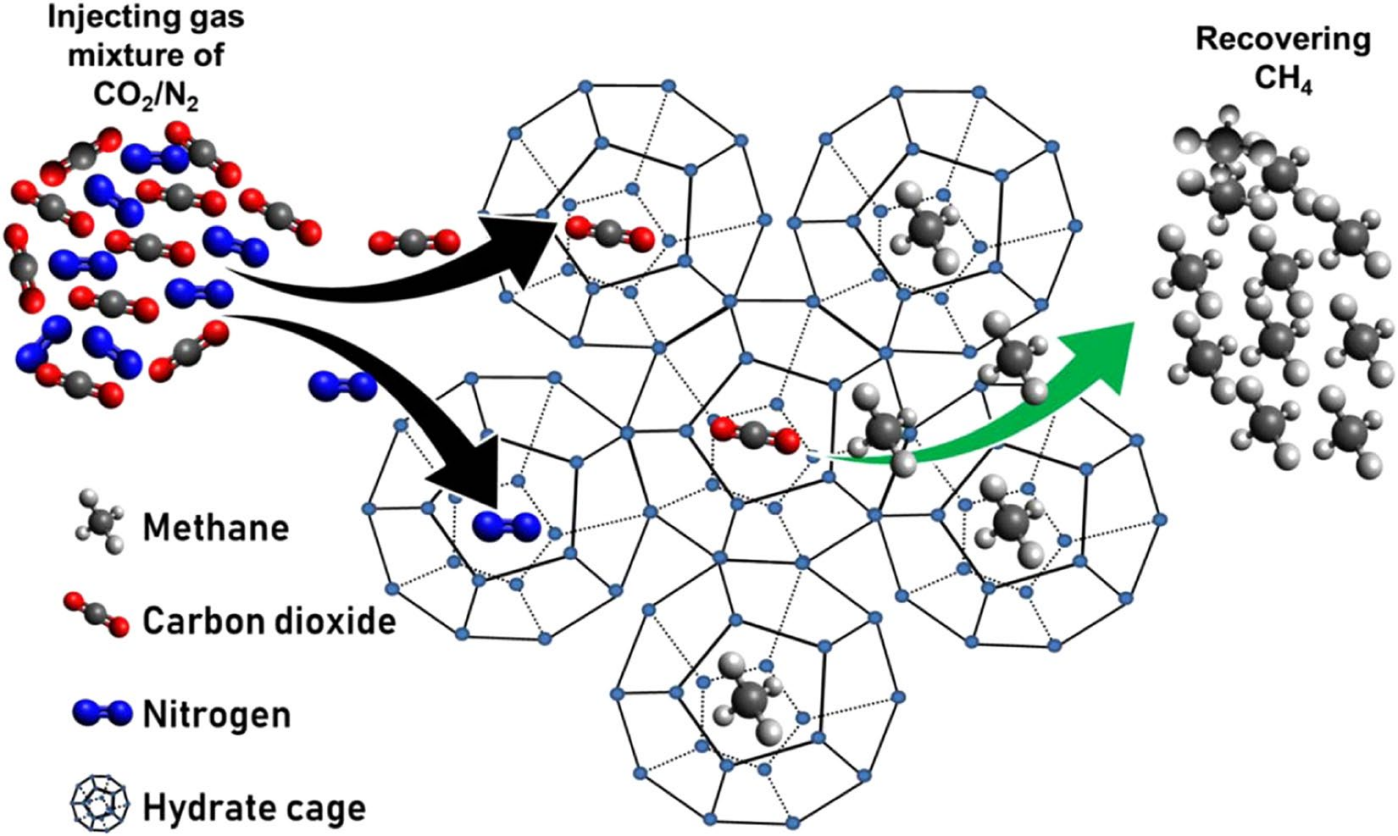
Schematic of the CH_4_-CO_2_/N_2_ replacement mechanism in naturally occurring hydrates.

To model this complex mechanism, as portrayed in Fig. 2, one needs to consider a twofold role of CH_4_ involved in the dissociation and reformation of the gas hydrates. Herein, as a preliminary approach, we are formulating the CH_4_ displacement rate during the gas exchange process as,

9 $$[\begin{array}{c}{\rm{Displacement}}\,{\rm{of}}\,{{\rm{CH}}}_{4}\\ {\rm{during}}\,{\rm{replacement}}\,{\rm{process}}\end{array}]=[\begin{array}{c}{\rm{Dissociation}}\,{\rm{of}}\\ {{\rm{CH}}}_{4}\,{\rm{hydrate}}\end{array}]+[\begin{array}{c}{\rm{Formation}}\,{\rm{of}}\,{\rm{replacing}}\,{\rm{agents}}\\ ({\rm{pure}}\,{{\rm{CO}}}_{2}\,{\rm{or}}\,{\rm{mixture}}\,{\rm{of}}\,{{\rm{CO}}}_{2}\,{\rm{and}}\,{{\rm{N}}}_{2})\\ {\rm{and}}\,{{\rm{CH}}}_{4}\,{\rm{hydrates}}\end{array}]$$

Accordingly, the mathematical formulation is proposed as,

10 $$\frac{d{n}_{{{\rm{CH}}}_{4},{\rm{H}}}}{dt}=-\,{k}_{{\rm{d}}}{A}_{e,{\rm{d}}}\,{(\Delta \mu )}_{{{\rm{CH}}}_{4}}{n}_{{{\rm{H}}}_{2}{\rm{O}},{\rm{H}}}+\gamma \,{k}_{{\rm{f}}}{A}_{e,{\rm{f}}}\,{(\Delta \mu )}_{{\rm{RG}}}{n}_{{{\rm{H}}}_{2}{\rm{O}},{\rm{L}}}$$

where, suffixes f and d are used for the formation and decomposition of hydrate, respectively. RG is the replacing gas agent, such as pure CO_2_, mixture of CO_2_-N_2_, CO_2_-H_2_ etc. Now, *γ*, the ratio of the sum of fractional occupancy of the CH_4_ in small and large hydrate cages to that of the replacing gas is estimated as,

11 $$\gamma =\mathop{\sum }\limits_{i=1}^{2}{\theta }_{i,{{\rm{CH}}}_{4}}/\mathop{\sum }\limits_{i=1}^{2}{\theta }_{i,{\rm{RG}}}$$

in which, these fractional occupancies are evaluated from the Langmuir type expression, which is detailed elsewhere^28^.

With this concept, the replacement model is obtained as follows,

12 $$\begin{array}{c}{n}_{{{\rm{CH}}}_{4},{\rm{H}}}=[\frac{{f}_{{\rm{RG}}^{\prime\prime} }(\exp \,[({f}_{{{\rm{CH}}}_{4}}-{f}_{R{\rm{G}}^{\prime} })/C])\,(\exp \,[({f}_{{{\rm{CH}}}_{4}}+{f}_{R{\rm{G}}^{\prime} }-C)\,t]-1)}{({f}_{{{\rm{CH}}}_{4}}+{f}_{R{\rm{G}}^{\prime} }-C)\,\exp \,[({f}_{{{\rm{CH}}}_{4}}\exp \,(Ct)\,-{f}_{R{\rm{G}}^{\prime} }\exp \,(\,-\,Ct))/C]}]\\ \,\,\,+[\frac{{n}^{0}(\exp \,[({f}_{{{\rm{CH}}}_{4}}-{f}_{R{\rm{G}}^{\prime} })/C])}{\exp \,[({f}_{{{\rm{CH}}}_{4}}\exp \,(Ct)-{f}_{R{\rm{G}}^{\prime} }\exp \,(\,-\,Ct))/C]}]\end{array}$$

All the specific terms involved in the above equation are elaborated later (see ‘Methods’ section). This is the final representation of the proposed model to predict the transient behavior of CH_4_-CO_2_ (pure/mixed) replacement with porous media in the presence of salt water.

### Determining optimal model parameters

The proposed formulation includes total four parameters, namely *α*, *β*_0_, *C* and *k*_0_. They are inherently linked with the real-time occupancy of guest gases, involved surfaces of porous sediments, renewal in reaction area and rate constant, respectively. These parameters are optimized with the following methodology.

### Optimization problem

13 $$\mathop{{\rm{Minimize}}\,\varphi }\limits_{U}\,=\,{\rm{AARD}}\,( \% )=(\frac{100}{{n}_{{\rm{dp}}}})(\mathop{\sum }\limits_{i=1}^{n}|\frac{{n}_{{\rm{gg}}}(\exp )-{n}_{{\rm{gg}}}({\rm{pred}})}{{n}_{{\rm{gg}}}(\exp )}|)$$

Subject to constraints:

14 $$0\le \alpha \le 1$$

15 $$0\le {\beta }_{0}\le 1$$

As shown, the objective function is concerned with the absolute average relative deviation (AARD) of the proposed model predictions (pred) from the actual/experimental (exp) data. *U* combines a set of optimization parameters as,

16 $$U=[\begin{array}{c}\alpha \\ {\beta }_{0}\\ C\\ {k}_{0}\end{array}]$$

The objective function is minimized with respect to the proposed model parameters *α*, *β*_0_, *k*_0_ and *C*. For this, we use a nonlinear programming method, namely generalized reduced gradient (GRG). In this method, the nonlinear objective function is linearized at a local solution through the Taylor series expansion. The optimal solution reaches when the partial derivative of an objective function with respect to decision variables is equal to zero^33^. The GRG method is only one of a class of algorithms based on implicit variable elimination. This process begins with the evaluation of reduced gradient vector $\nabla \tilde{f}$ as,

17 $$\nabla \tilde{f}=\nabla \tilde{f}({x}^{(t)})-\nabla \hat{f}({x}^{(t)})\,{{\rm{J}}}^{-1}{\rm{C}}$$

where, *t* is the iteration, J and C the constraint gradient sub-matrix of basic ($\hat{x}$) and non-basic variables ($\bar{x}$). If $\Vert \nabla \tilde{f}\Vert \ge \varepsilon$, then process terminates, otherwise one has to set descent direction (dd) as: ${\rm{dd}}=(\hat{{\rm{d}}},\,\bar{{\rm{d}}})=[\,-{{\rm{J}}}^{-1}{\rm{C}}\,\bar{{\rm{d}}},$ ${(-\nabla \bar{f})}^{{\rm{T}}}]$ and minimize $f({x}^{(t)}+\omega {\rm{d}})$ with respect to scalar parameter *ω*. This nonlinear optimization is proposed to perform effortlessly in the solver tool of Microsoft Excel 2010.

### Case study: 2011–2012 Iġnik Sikumi field test

The prediction potential of the proposed formulation will be analysed by evaluating its performance to predict the actual field scale data. For this, we consider the Iġnik Sikumi field test conducted in Alaska North Slope during 2011 and 2012^34^. The primary objective of this project was to investigate the feasibility and real-time performance of the CH_4_-CO_2_/N_2_ gas exchange dynamics into the actual hydrate bearing reservoirs. In this test, the injection and production phases are performed through a single vertical well from the temporary ice-pad situated in Prudhoe Bay Unit L-pad. This scheme is usually referred to “huff and puff” method. The process comprises of two principal operational phases: (i) injecting/introducing highly recognized and effective replacing gas mixture of CO_2_ (23 mol%) and N_2_ (77 mol%) into the reservoir, and (ii) producing CH_4_ and recovering CO_2_ and N_2_ with and without any assistance from the same reservoir. This process was started with the injection of the aforesaid replacing agent at a pressure and temperature of 9.8 MPa and 278.15 K, respectively into the reservoir for 14 days, which is followed by 2.5 days of shut-in soak period. During these 2.5 days, the bottom-hole pressure is reduced from 9.8 to 8.27 MPa. Then, the production/flowback phase was conducted in four stages as,

- Stage 1 – Unassisted flowback for 1.5 days
- Stage 2 – Jet-pump assisted flowback with *P*_production_ > *P*_MH stability_ for 9 days
- Stage 3 – Jet-pump assisted flowback with ${P}_{{\rm{production}}}\cong {P}_{{\rm{MH}}{\rm{stability}}}$ for 2.5 days
- Stage 4 – Jet-pump assisted flowback with *P*_production_ < *P*_MH stability_ for 18.5 days

Basically, this production operation was carried out into two broad parts i.e., unassisted (1.5 days) and jet-pump assisted (30 days) flowback with the proper shut-in period for repairing the associated equipment. Further, the jet-pump assisted phase is divided into three stages by manipulating the production/well pressure (*P*_production_) with reference to the methane hydrate stability pressure (*P*_MH stability_). The detailed experimental observations associated with this field test are open to public^35^.

### Performing simulation experiment

**Figure 3:**
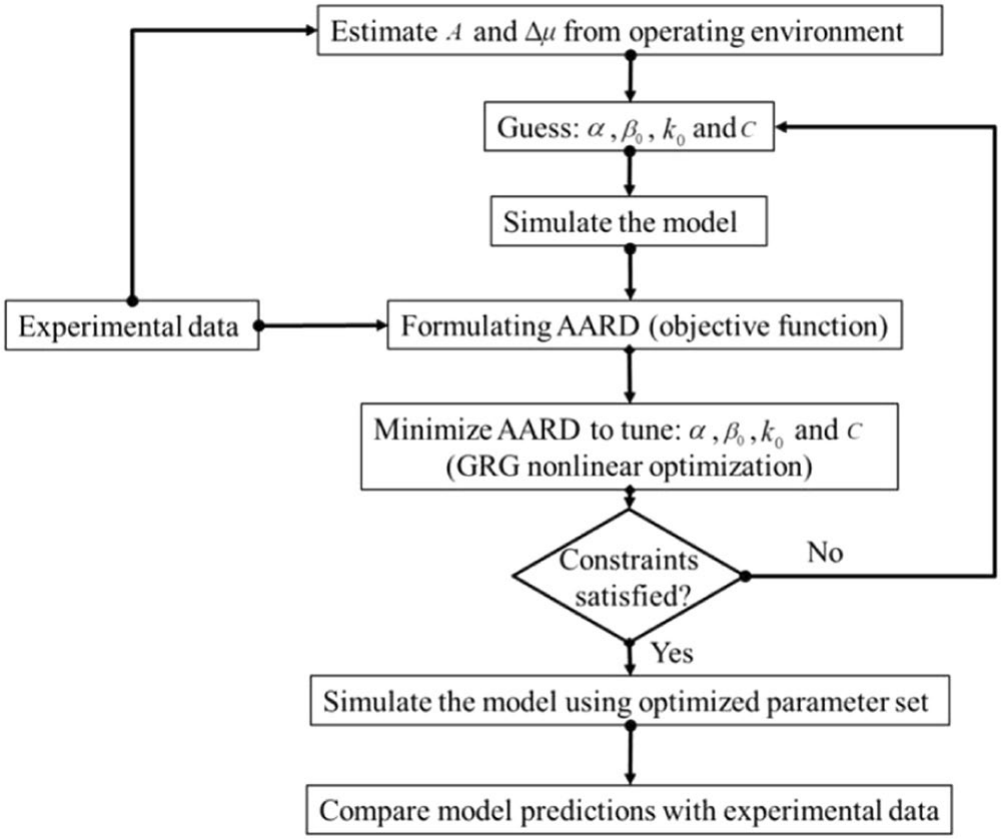
Sequential algorithm for the simulation experiment of the proposed formulation.

We aim to simulate the proposed formulation to interpret the production behavior of the guest gases (CH_4_, CO_2_ and N_2_) involved in the operation of all the stages. For this, we have developed a sequential computer-assisted algorithm as outlined in Fig. 3.

At first, all the components of the proposed formulation need to be analysed before their combined evaluation. They mainly include the total reaction surface area of the involved porous sediment and the driving force of the operation.

### Evaluating reaction surface area

The well-log analysis made based on the well log and mud log data confirmed^34^ that the gas hydrates are present in close proximity to the Prudhoe Bay unit L-pad. Further, the geophysical reservoir characterization of the L-106 well confirmed the presence of four different hydrate bearing sandstone sediments namely, C-2, C-1, D and E. Among these, the homogeneous, thick and highly CH_4_ hydrate saturated sediment (i.e., “C-1 sand”) has been selected as a primary testing zone. This layer is located in the range of 683.67–692.81 m (2,243–2,273 ft) below the sea level and has 40% porosity, 72% average gas hydrate saturation (*S*_H_) and 28% water saturation (*S*_w_). This region typically has the pressure and temperature of 6.9 MPa and 278.15 K, respectively with the formation breakdown pressure of 10 MPa. For this hydrate bearing sediment, the surface area of the unconsolidated irregular porous sediment is estimated from Eq. (2). In that equation, the main challenge is to estimate the total volume of the porous material (*V*_Tp_) available in this testing zone. For this, we use the value of the void volume and porosity of the bed. Among these, the porosity of the concerned sediment is available^34^. Thus, the accuracy of the evaluated reaction surface is exclusively dependent on the porosity of the bed (here, 40%). Furthermore, the void volume of the bed is projected from the average gas hydrate saturation (here, 72%) and the total volume of methane hydrates (*V*_T,MH_) across the bed as: *V*_void_=*V*_T,MH_/*S*_H_, in which, *V*_T,MH_ is calculated from the total volume of the recovered methane, assuming that almost all the methane hydrates present in the testing zone is recoverable.

### Assessing driving force

The next important component of the proposed formulation is the driving force. Here, we use the difference in chemical potentials of water in hydrate and liquid phase as a driving force, which is defined in Eq. (3). There are two major components involved, namely ${\mu }_{{\rm{w}}}^{{\rm{H}}}$ and ${\mu }_{{\rm{w}}}^{{\rm{L}}}$ [Eqs. (4) and (5)]. Among these, ${\mu }_{{\rm{w}}}^{{\rm{H}}}$ is a function of the fractional occupancy of the involved guest gas. This fractional occupancy further depends on the fugacity of the guest gas and the Langmuir constant. Here, the temperature dependent Langmuir constant is calculated from the Kihara type potential parameters and the detailed procedure is available elsewhere^22^. Here, the Kihara potential parameters for CH_4_, CO_2_ and N_2_ include: (i) *a* (Å) equals 0.3834, 0.6805 and 0.6805, respectively, (ii) σ (Å) equals 3.14393, 2.97638 and 3.13512, respectively, and (iii) *ε*/*K* (K) equals 155.593, 175.405 and 127.426, respectively^36^. Now, in the course of four production phases, the well pressure fluctuates, and consequently, the fugacity of each component in the produced gas mixture gets altered, which then affects the hydrate cage occupancy of the involved guest gases - CH_4_, CO_2_ and N_2_. Thus, the hydrate phase chemical potential of water changes during the flowback phases. For this, we have evaluated the fugacities of all those three guest gases from the Soave-Redlich-Kwong (SRK) equation of state^37^ at each time step using the indigenously developed MATLAB code.

The chemical potential of water in the liquid phase comprises of $\Delta {h}_{{\rm{w}}}^{{\rm{L}}}$, $\Delta {V}_{{\rm{w}}}^{{\rm{L}}}$ and *a*_w_, which are the function of operating temperature and well pressure. Like the fractional occupancy, these components are evaluated at each time step for the fluctuating well pressure and at the operating temperature. Importantly, *a*_w_ is computed by accounting the practical issues associated with the two principal constituents (porous sand sediment and seawater) of the marine hydrate reservoirs. As far as the current case study is concerned, the hydrate bearing unit has 72% CH_4_ hydrates and 28% water (9% free and 19% bound), leaving no room for the free gases. Naturally, a very less amount of water is available to associate with the injected guest gases to form the new hydrates, and there will be no alternative for the injected CO_2_-N_2_ (23–77 mol%) other than following the direct gas exchange mechanism. Besides, from the well log data, the quartz is perceived as a dominant reservoir mineral, and there was no significant mineral dissolution or precipitation of the reservoir minerals into the liquid phase (water) during the operation^16^. Thus, for this case, it is not reasonable to consider their contribution during the simulation run. For simulating the present case reservoir data, the influence of associated water-guest gases and porous material is taken into account.

### Estimating model parameters

Once the calculation of *A* and Δ*μ* is over, we proceed to estimate the model parameters i.e., *α*, *β*_0_, *k*_0_ and *C*, as mentioned in Fig. 3. We start with the guess value of the tuning parameters and simulate the proposed model to predict the moles of CH_4_, CO_2_ and N_2_ gases recovery during the production stages. Afterward, the deviation of the predicted response from the field data is used to find the absolute average relative deviation (AARD). We set this AARD as an objective function for the nonlinear optimization problem subject to the constraints stated before. As mentioned, for this, we have used the generalized reduced gradient nonlinear optimization method in the Solver tool inbuilt in Microsoft Excel 2010 setup. This robust optimization technique takes a few seconds to optimize the extensive production data of 31.5 days (unassisted and jet-pump assisted flowbacks). Values of the optimal model parameters are placed in Table 1. The proposed formulation can easily be used for online tuning at diverge geographical conditions.

**Table 1:** Parameter sets of the proposed formulation.

Gas	Stage no.	Parameter			
		${K}_{0}^{{\rm{a}}}$ × 10^−6^	*α*	*β*_0_	*C* × 10^2^
CH_4_	1	19.6267	0.2283	0.5744	1.0420
	2	170.3098	0.2065	0.6664	0.0150
	3	77.1812	0.1867	0.6815	0.0900
	4	8.6874	0.0897	0.9009	0.0055
CO_2_	1	4.5542	0.1748	0.9108	0.4000
	2	5.4967	0.2064	0.9302	0.0004
	3	3.3693	0.1808	0.7401	0.0600
	4	0.7930	0.2160	0.8191	0.0034
N_2_	1	4.6051	0.1680	0.9210	0.4000
	2	4.4630	0.1913	0.8926	0.0004
	3	4.3953	0.2054	0.8825	0.0400
	4	1.5479	0.2411	0.7917	0.0010

^a^mol of guest gas^1^ mol of H_2_O^-1^ m^−2^ min^−1^.

### Predicting field data

**Figure 4:**
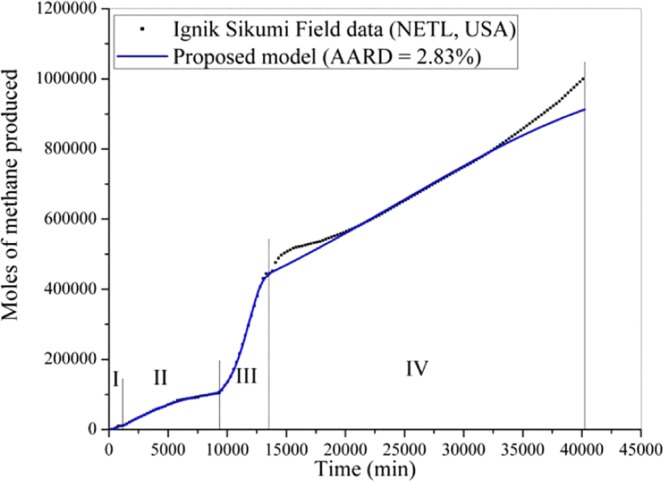
Performance of the proposed formulation in predicting the Iġnik Sikumi field data for methane gas recovery.

**Figure 5:**
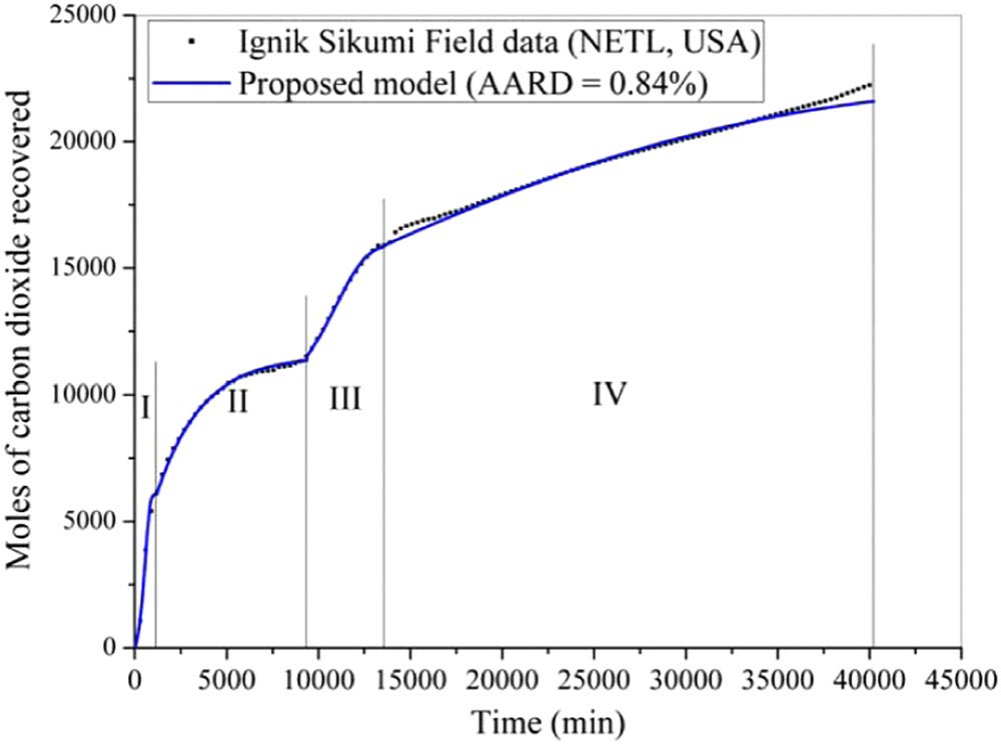
Performance of the proposed formulation in predicting the Iġnik Sikumi field data for carbon dioxide gas recovery.

**Figure 6:**
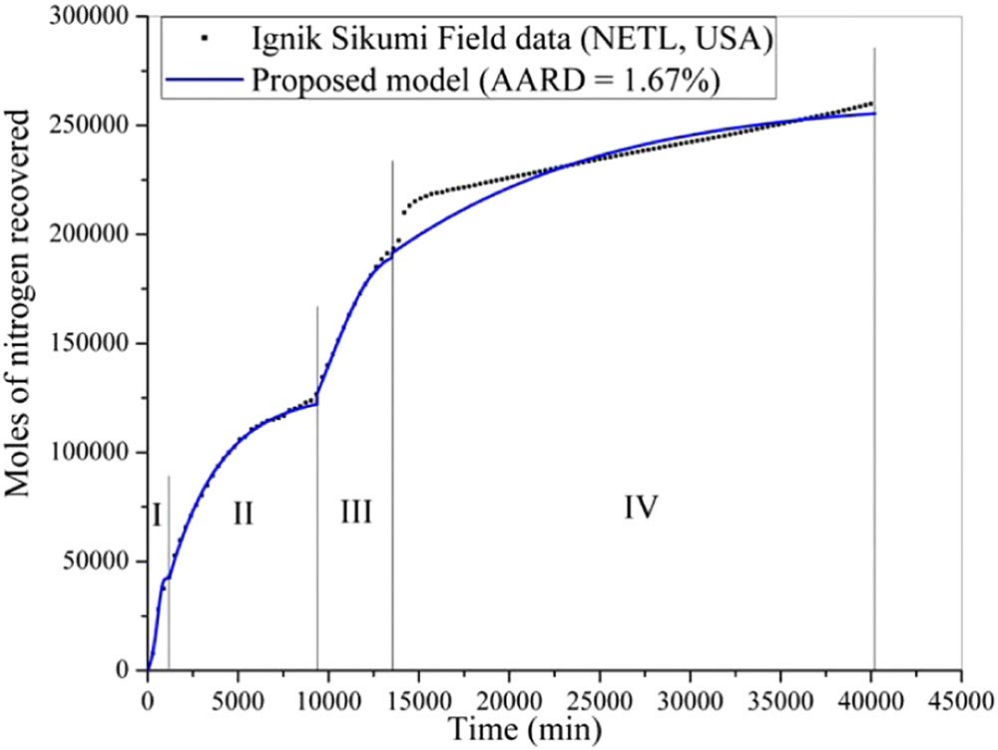
Performance of the proposed formulation in predicting the Iġnik Sikumi field data for nitrogen gas recovery.

In this section, the proposed formulation is employed to predict the Iġnik Sikumi field data for natural gas recovery with carbon dioxide sequestration. For this, Figs. 4–6 are produced to investigate the model performance in terms of the dynamic production profile of CH_4_, CO_2_ and N_2_, respectively during the four production phases. In this operation^34^, total 215.9 Mscf (6,113.6 m^3^) of the mixture of replacing agents (CO_2_-N_2_) was introduced into 9.14 m (30 ft) thick C-1 sand of L-106 bore well at the injection pressure and temperature of 9.8 MPa and close to 278.15 K, respectively. This gas mixture includes 77% [i.e., 167.3 Mscf (4,737.4 m^3^)] of N_2_ and 23% [i.e., 48.6 Mscf (1,376.2 m^3^)] of CO_2_. The injection phase continued for 14 days, during which, the carbon dioxide and nitrogen were transforming themselves from gaseous phase to hydrate phase by the replacement of encaged methane existed in the hydrate phase. As a result, during the flowback phases of this operation, 855 Mscf (24,410 m^3^) of CH_4_ gas was produced, while 29.16 Mscf (825.72 m^3^) and 50.19 Mscf (1,421.22 m^3^) of CO_2_ and N_2_ were retained in the reservoir, respectively^34^. The recovery efficiency (*η*_rec_) defined by the following formula,

18 $${\eta }_{{\rm{rec}}}\,( \% )=\frac{{V}_{{\rm{gg}},\uparrow }}{{V}_{{\rm{gg}},\downarrow }}\times 100$$

is 40% for CO_2_ and 70% for N_2_. Basically, the major portion of the carbon dioxide has been successfully sequestrated into the well. Here, ${V}_{{\rm{gg}},\uparrow }$ and ${V}_{{\rm{gg}},\downarrow }$ are the volume of guest gas (such as CH_4_, CO_2_ and N_2_) recovered from and injected into the well, respectively.

At the start of the production phase, for the unassisted flowback (stage 1–1.5 days), the CH_4_ gas recovery rate is slow, and it has dominated the composition in the produced gas mixture. In the second stage (~9 days), the composition of CH_4_ in the gas mixture further increases and it reaches around 80 mol% of the total gas produced during this period. Then, for the third (~2.5 days) and fourth stages (~18.5 days), the composition of CH_4_ goes on swelling (up to 95 mol%) in the produced gas mixture, while CO_2_ and N_2_ share a very little contribution. In fact, CO_2_ has never exceeded 2 mol% of the total produced stream of the gas mixture^34^. For predicting these outcomes, we use the model having a set of optimized parameters provided in Table 1. With this, the proposed formulation predicts the CH_4_, CO_2_ and N_2_ gases recovery with only 2.83, 0.84 and 1.67% AARD, respectively. From the prediction accuracy, it becomes obvious that the proposed model is rigorous and versatile enough.

### Predicting laboratory scale data

#### Hydrate formation

**Figure 7:**
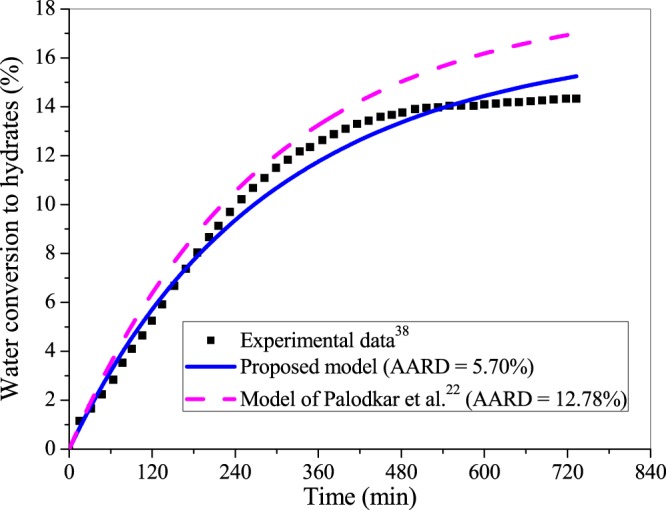
Comparing the performance of the proposed framework and existing model^22^ with reference to the experimental data^38^ of water conversion to methane hydrate in the presence of silica sand with 100% water saturation at operating pressure and temperature of 5.75 MPa and 274.5 K, respectively.

To investigate the versatility of the proposed formulation, we further compare the performances of the proposed and existing^22^ model for the laboratory scale experimental data of CH_4_ hydrate kinetics. Figure 7 depicts the comparative performance with reference to the experimental data^38^ of water conversion to CH_4_ hydrates in the bed of silica sand with 100% water saturation at operating pressure and temperature of 5.75 MPa and 274.5 K, respectively. This setup (Fig. S1) is presented in the supplementary information file and optimal model parameters are listed in Table S1. It is evident that the proposed model performs better than the existing model^22^, which is also obvious from the respective AARD (%) values reported in Fig. 7. This is because the proposed model takes into account many practical aspects involved in hydrate dynamics.

**Figure 8:**
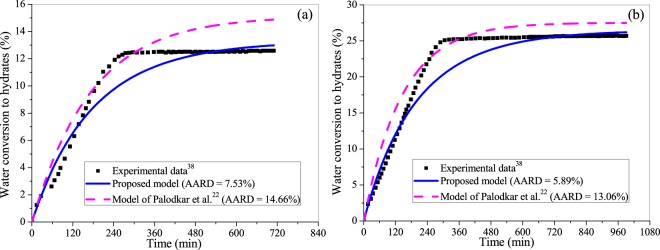
Comparing the performance of the proposed framework and existing model^22^ with reference to the experimental data^38^ of water conversion to methane hydrate in the presence of porous bed (50% silica sand and 50% clay) with (**a**) 75% and (**b**) 50% water saturation at operating pressure and temperature of 6.10 MPa and 274.5 K, respectively.

Further, we evaluate the proposed model for the CH_4_ hydrate growth in the mixture of silica sand and clay. Figure 8(a,b) compare the hydrate growth in the bed of 50% silica sand and 50% clay with 75% and 50% water saturation, respectively. In both the cases, the operating pressure and temperature are 6.10 MPa and 274.5 K, respectively. It is seen in Fig. 8 that the proposed model predicts the CH_4_ hydrate growth better than the existing model for the reason stated above. This observation is confirmed by the percent AARD values provided in the same figure.

#### Hydrate dissociation

**Figure 9:**
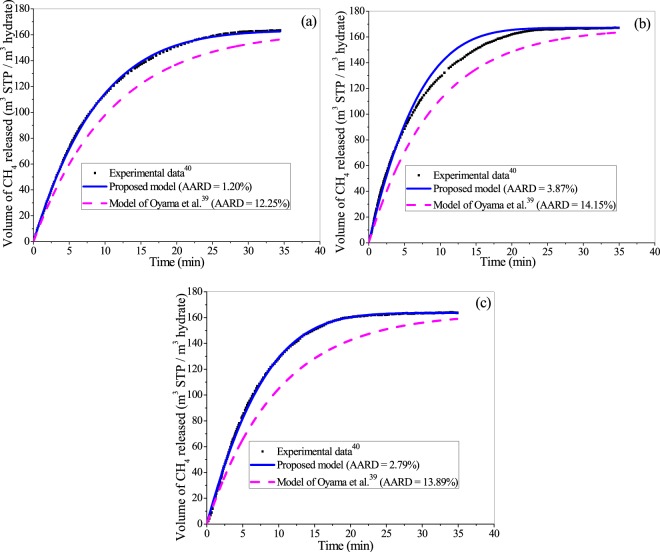
Comparing the performance of the proposed formulation and existing model39 with reference to the experimental data40 of water conversion to methane hydrate in the presence of silica sand bed (75% saturation) and (**a**) pure water, and (**b**) 1.5 wt% and (**c**) 3.0 wt% of aqueous salt solution at operating pressure and temperature of 4.8 MPa and 297.2 K, respectively.

Figure 9(a–c) evaluate the performance of the proposed framework comparing with that of the existing model^39^ with reference to the experimental data^40^ of CH_4_ hydrate dissociation in the presence of silica sand, along with pure water, and 1.5 wt% and 3.0 wt% salt solution, respectively. In all these three cases, the operating pressure and temperature are 4.8 MPa and 297.2 K, respectively. The experimental setup is briefly presented in the supplementary file (Fig. S2). The optimal model parameters are documented in Table S2. For all the cases, the proposed model outperforms the existing one^39^, which is quantified through the AARD values reported in Fig. 9. This is because the proposed model has addressed several practical issues related to the porous media and saline water.

## Conclusion

This work proposes a theoretical formulation to portray the gas hydrate formation, decomposition and replacement dynamics in the actual hydrate reservoirs. This rigorous model formulates the two-fold CH_4_-CO_2_ (pure/mixed) exchange mechanism, addressing several practical aspects specific to the basic elements (porous media and saline water) of the hydrate reservoirs. Potential of the proposed framework is analysed by predicting the amount of CH_4_ produced, and CO_2_ and N_2_ recovered in the Iġnik Sikumi field test conducted on the Alaska North Slope in 2011 and 2012. This field test starts with the injection of CO_2_-N_2_ (23–77 mol%) gas mixture into the well for 14 days, which is then followed by 31.5 days of CH_4_ production and CO_2_-N_2_ recovery from the same bore well. For this, we perform the simulation experiments with the conditions at which the field scale experiment was exactly conducted. Thanks to the nonlinear optimization technique, using which, we have identified the optimized parameter set online for all the production phases. With this, the model has shown a promising performance in predicting the field scale data. It is also quantified based on the percent AARD obtained for CH_4_, CO_2_ and N_2_ as 2.83, 0.84 and 1.67, respectively with reference to the Iġnik Sikumi field data. To show the versatility of the proposed framework, further it is successfully tested with the laboratory scale data for CH_4_ hydrate formation and decomposition in the presence of pure and salt water, and porous media. This approves the online use of the proposed theory to interpret the actual field scale exploration of the naturally occurring hydrates through the replacement technique.

## Methods

### Modeling

The amount of water associated with the hydrate and liquid phase is expressed in terms of *n*_CH4,H_, η_H_ and *n*_RG,H_. With this, Eq. (10) is rewritten as,

19 $$\begin{array}{c}\frac{d{n}_{{{\rm{CH}}}_{4},{\rm{H}}}}{dt}+\{[{k}_{{\rm{d}}}{\beta }_{0}\exp \,(Ct)A\,{(\frac{{\mu }_{{\rm{w}}}^{{\rm{L}}}}{RT}-\frac{{\mu }_{{\rm{w}}}^{{\rm{H}}}}{RT})}_{C{H}_{4}}+\gamma \,{k}_{{\rm{f}}}{\beta }_{0}\exp \,(\,-\,Ct)\,A\,{(\frac{{\mu }_{{\rm{w}}}^{{\rm{H}}}}{RT}-\frac{{\mu }_{{\rm{w}}}^{{\rm{L}}}}{RT})}_{{\rm{RG}}}]\,({n}_{{\rm{H}}})\}\,{n}_{{{\rm{CH}}}_{4},{\rm{H}}}\\ \,\,\,=\{+\gamma \,{k}_{{\rm{f}}}{\beta }_{0}\exp \,(\,-\,Ct)\,A\,{(\frac{{\mu }_{{\rm{w}}}^{{\rm{H}}}}{RT}-\frac{{\mu }_{{\rm{w}}}^{{\rm{L}}}}{RT})}_{{\rm{RG}}}({n}_{{{\rm{H}}}_{2}O,{\rm{T}}})\}\end{array}$$

The above equation is simplified to,

20 $$\frac{d{n}_{{{\rm{CH}}}_{4},{\rm{H}}}}{dt}+P(t)\,{n}_{{{\rm{CH}}}_{4},{\rm{H}}}=Q\,(t)$$

in which, the time dependent parameters *P* and *Q* have the following representation,

21 $$P\,(t)=\{[{k}_{{\rm{d}}}{\beta }_{0}\exp \,(Ct)A{(\frac{{\mu }_{{\rm{w}}}^{{\rm{L}}}}{RT}-\frac{{\mu }_{{\rm{w}}}^{{\rm{H}}}}{RT})}_{{{\rm{CH}}}_{4}}+\gamma \,{k}_{{\rm{f}}}{\beta }_{0}\exp \,(\,-\,Ct)\,A{(\frac{{\mu }_{{\rm{w}}}^{{\rm{H}}}}{RT}-\frac{{\mu }_{{\rm{w}}}^{{\rm{L}}}}{RT})}_{{\rm{RG}}}]({n}_{{\rm{H}}})\}$$

22 $$Q\,(t)=\{+\gamma \,{k}_{{\rm{f}}}{\beta }_{0}\exp \,(\,-\,Ct)\,A\,{(\frac{{\mu }_{{\rm{w}}}^{{\rm{H}}}}{RT}-\frac{{\mu }_{{\rm{w}}}^{{\rm{L}}}}{RT})}_{{\rm{RG}}}({n}_{{{\rm{H}}}_{2}O,{\rm{T}}})\}$$

Now, the aforementioned differential Eq. (20) is solved by the use of the integrating factor method. The integrating factor (IF) is defined as,

23 $${\rm{IF}}=\exp \,\int P\,dt=\exp \,\{[{k}_{{\rm{d}}}{\beta }_{0}A{(\frac{{\mu }_{{\rm{w}}}^{{\rm{L}}}}{RT}-\frac{{\mu }_{{\rm{w}}}^{{\rm{H}}}}{RT})}_{{{\rm{CH}}}_{4}}(\frac{\exp \,(Ct)}{C})+\gamma \,{k}_{{\rm{f}}}{\beta }_{0}A{(\frac{{\mu }_{{\rm{w}}}^{{\rm{H}}}}{RT}-\frac{{\mu }_{{\rm{w}}}^{{\rm{L}}}}{RT})}_{{\rm{RG}}}(\frac{\exp \,(\,-\,Ct)}{-C})]({n}_{{\rm{H}}})\}$$

Multiplying IF in both sides of Eq. (20),

24 $$({\rm{IF}})\frac{d{n}_{{{\rm{CH}}}_{4},{\rm{H}}}}{dt}+({\rm{IF}})\,P(t)\,{n}_{{{\rm{CH}}}_{4},{\rm{H}}}=({\rm{IF}})\,Q\,(t)$$

The general solution of the above equation is,

25 $${\rm{IF}}\,{n}_{{{\rm{CH}}}_{4},{\rm{H}}}=\int ({\rm{IF}})\,Q\,(t)\,dt$$

The right hand side (RHS) of this equation is evaluated as,

26 $$\begin{array}{c}{\rm{RHS}}=\int \exp \,\{[{k}_{{\rm{d}}}{\beta }_{0}A{(\frac{{\mu }_{{\rm{w}}}^{{\rm{L}}}}{RT}-\frac{{\mu }_{{\rm{w}}}^{{\rm{H}}}}{RT})}_{{{\rm{CH}}}_{4}}(\frac{\exp \,(Ct)}{C})+\gamma \,{k}_{{\rm{f}}}{\beta }_{0}A{(\frac{{\mu }_{{\rm{w}}}^{{\rm{H}}}}{RT}-\frac{{\mu }_{{\rm{w}}}^{{\rm{L}}}}{RT})}_{{\rm{RG}}}(\frac{\exp \,(\,-\,Ct)}{-C})]({n}_{{\rm{H}}})\}\\ \,\,\,\,\{[+\gamma \,{k}_{{\rm{f}}}{\beta }_{0}\exp \,(\,-\,Ct)\,A{(\frac{{\mu }_{{\rm{w}}}^{{\rm{H}}}}{RT}-\frac{{\mu }_{{\rm{w}}}^{{\rm{L}}}}{RT})}_{{\rm{RG}}}({n}_{{{\rm{H}}}_{2}O,{\rm{T}}})]\}\,dt\end{array}$$

Expressing exponential term present in the above equation by the use of the Taylor series expansion up to first order of time,

27 $$\begin{array}{c}{\rm{RHS}}=[+\gamma \,{k}_{{\rm{f}}}{\beta }_{0}\,A{(\frac{{\mu }_{{\rm{w}}}^{{\rm{H}}}}{RT}-\frac{{\mu }_{{\rm{w}}}^{{\rm{L}}}}{RT})}_{{\rm{RG}}}({n}_{{{\rm{H}}}_{2}O,{\rm{T}}})]\times \\ \int \exp \{[(\frac{{k}_{{\rm{d}}}{\beta }_{0}A\,{({\mu }_{{\rm{w}}}^{{\rm{L}}}-{\mu }_{{\rm{w}}}^{{\rm{H}}})}_{{{\rm{CH}}}_{4}}}{RTC})(1+Ct)+(\frac{\gamma \,{k}_{{\rm{f}}}{\beta }_{0}A\,{({\mu }_{{\rm{w}}}^{{\rm{H}}}-{\mu }_{{\rm{w}}}^{{\rm{L}}})}_{{\rm{RG}}}}{-RTC})(1-Ct)]({n}_{{\rm{H}}})\}\,\exp \,(\,-\,Ct)\,dt\end{array}$$

Considering,

28 $$\begin{array}{c}{f}_{{{\rm{CH}}}_{4}}={k}_{{\rm{d}}}{\beta }_{0}A\,{(\frac{{\mu }_{{\rm{w}}}^{{\rm{L}}}}{RT}-\frac{{\mu }_{{\rm{w}}}^{{\rm{H}}}}{RT})}_{{{\rm{CH}}}_{4}}{n}_{{\rm{H}}}\\ {f}_{R{\rm{G}}^{\prime} }=\gamma \,{k}_{{\rm{f}}}{\beta }_{0}A\,{(\frac{{\mu }_{{\rm{w}}}^{{\rm{H}}}}{RT}-\frac{{\mu }_{{\rm{w}}}^{{\rm{L}}}}{RT})}_{{\rm{RG}}}{n}_{{\rm{H}}}\\ {f}_{{\rm{RG}}^{\prime\prime} }=\,\gamma \,{k}_{{\rm{f}}}{\beta }_{0}\,A\,{(\frac{{\mu }_{{\rm{w}}}^{{\rm{H}}}}{RT}-\frac{{\mu }_{{\rm{w}}}^{{\rm{L}}}}{RT})}_{{\rm{RG}}}{n}_{{{\rm{H}}}_{2}O,{\rm{T}}}\end{array}\}$$

Equation (27) becomes,

29 $${\rm{RHS}}={f}_{{\rm{RG}}^{\prime\prime} }\int \exp [(\frac{{f}_{{{\rm{CH}}}_{4}}}{C})(1+Ct)-(\frac{{f}_{R{\rm{G}}^{\prime} }}{C})(1-Ct)]\exp \,(\,-\,Ct)\,dt$$

Integrating the above equation, we substitute that into Eq. (25) and get

30 $${\rm{IF}}\,{n}_{\rm{CH_4}{\rm{H}}}=\left[\frac{{f}_{{\rm{RG}}^{\prime\prime} }}{({f}_{{{\rm{CH}}}_{4}}+{f}_{R{\rm{G}}^{\prime} }-C)}\right][\exp (\frac{{f}_{{{\rm{CH}}}_{4}}-{f}_{R{\rm{G}}^{\prime} }}{C})]\,[\exp [({f}_{{{\rm{CH}}}_{4}}+{f}_{R{\rm{G}}^{\prime} }-C)t]]+{\rm{IC}}$$

Since at *t* = 0, n_gg,H_ = n^0^, the above equation yields,

31 $${\rm{IC}}=({{\rm{IF}}}^{0}\,{n}^{0})-[\frac{{f}_{{\rm{RG}}^{\prime\prime} }}{({f}_{{{\rm{CH}}}_{4}}+{f}_{R{\rm{G}}^{\prime} }-C)}\times \exp (\frac{{f}_{{{\rm{CH}}}_{4}}-{f}_{R{\rm{G}}^{\prime} }}{C})]$$

Inserting Eq. (31) into (30), dividing both sides by IF and simplifying,

32 $$\begin{array}{c}{n}_{{{\rm{CH}}}_{4},H}=[\begin{array}{c}(\frac{{f}_{{\rm{RG}}^{\prime\prime} }}{{\rm{IF}}\,({f}_{{{\rm{CH}}}_{4}}+{f}_{R{\rm{G}}^{\prime} }-C)})\times (\exp \,[({f}_{{{\rm{CH}}}_{4}}-{f}_{R{\rm{G}}^{\prime} })/C])\\ \,\times (\exp \,[({f}_{{{\rm{CH}}}_{4}}+{f}_{R{\rm{G}}^{\prime} }-C)t]-1)\end{array}]\\ \,\,\,+[\frac{{n}^{0}(\exp \,[({f}_{{{\rm{CH}}}_{4}}-{f}_{R{\rm{G}}^{\prime} })/C])}{{\rm{IF}}}]\end{array}$$

## Supplementary information

Supplementary information
