# Supplementary material for: Modeling recovery of natural gas from hydrate reservoirs with carbon dioxide sequestration: Validation with Iġnik Sikumi field data
Source: Sci Rep. 2019 Dec 11;9:18901. doi: 10.1038/s41598-019-55476-1 (PMC6906469; doi:10.1038/s41598-019-55476-1)
Supplement: Supplementary file 1 — Supplementary information [file 41598_2019_55476_MOESM1_ESM.pdf]

Supplementary Material

Modeling recovery of natural gas from hydrate reservoirs with carbon dioxide sequestration: Validation with Iġnik Sikumi field data

Avinash V. Palodkar[1] and Amiya K. Jana[1,*]

Experimental arrangement 1 (ref 38)

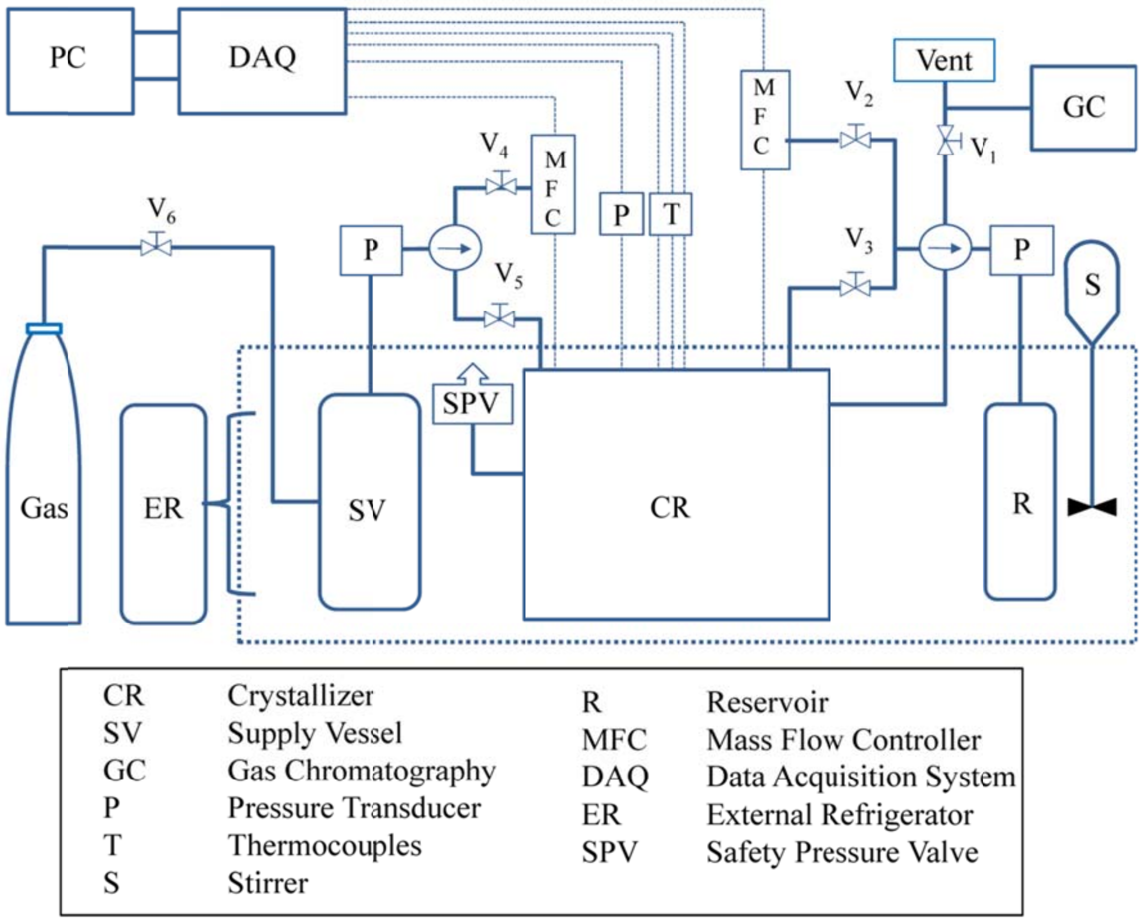

Fig. S1. Schematic of the experimental setup[38].

[1]Energy and Process Engineering Laboratory, Department of Chemical Engineering, Indian Institute of Technology, Kharagpur, India–721302.
[*]Corresponding author. Email: akjana@che.iitkgp.ac.in

Fig. S1 presents the schematic of an experimental setup employed for pure CH_4 hydrate formation in the presence of silica sand with particle size distribution of 30-400 μm. The crystallizer (SS-316) is equipped with a WIKA pressure transducer (0–20 MPa), along with a Bourdon pressure gauge to know the pressure inside the reactor. A set of mass flow meter and constant pressure controller (MFC) is there with a data acquisition system to measure and record the flow rate and pressure inside the reactor. For temperature measurement, PT-100 RTDs are inserted inside the crystallizer. The temperature along with the pressure information are recorded through the data acquisition system connected to a computer.

Experimental arrangement 2 (ref 40)

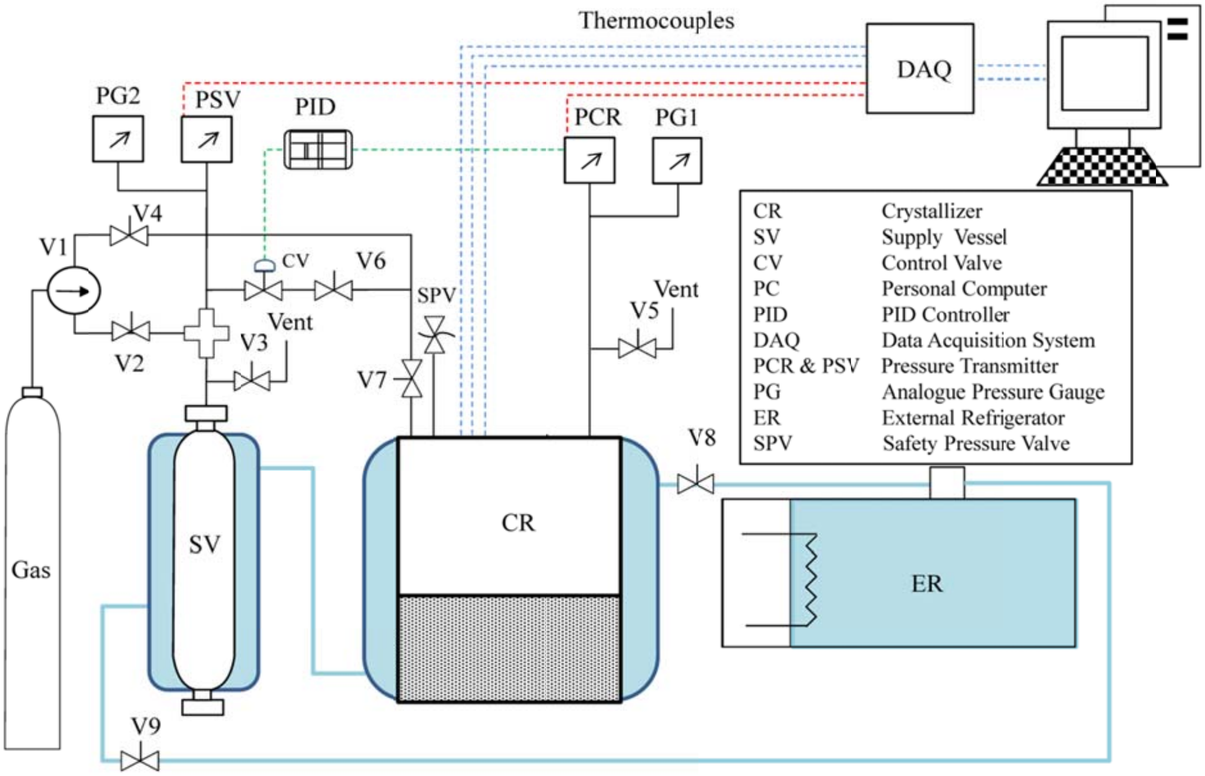

Fig. S2. Schematic of the experimental setup[40].

The experimental setup comprises of (i) a crystallizer made of stainless steel (SS 316), with an inner diameter of 10.2 cm and inner height of 26.7 cm, (ii) a cooling jacket to control the reactor

temperature, (iii) a multipoint thermocouple (Omega copper-constantan T-type, ±0.1 K) to measure the temperature, (iv) an external refrigerator (ER) to maintain the temperature and (v) Rosemount smart pressure transmitter (±20 kPa) and a WIKA analog gauge. Here, the crystallizer and gas reservoir are connected to a control valve, which is coupled with a PID controller to maintain the constant pressure during the decomposition process. The temperature and pressure readings are picked up in a computer (PC) using the data acquisition system and LabView software.

Table S1. Optimal model parameters for CH_4 hydrate formation kinetics.

Case	Proposed model				Palodkar et al.[22]			
	α	K_0^{a} $\times 10^{-14}$	β_0	C	α	K_0^{a} $\times 10^{-8}$	ΔE_a^{b} $\times 10^{-3}$	β
CH_4 hydrate formation || Pure water || Silica sand || 100% water saturation || 5.75 MPa || 274.5 K	0.5762	1.4622	0.7311	0.0030	0.1834	8.0095	63.9068	0.6007
CH_4 hydrate formation || Pure water || 50% silica sand and 50% clay || 75% water saturation || 6.10 MPa || 274.5 K	0.5648	2.0038	0.8939	0.0051	0.1512	9.5992	75.9245	0.6199
CH_4 hydrate formation || Pure water || 50% silica sand and 50% clay || 50% water saturation || 6.10 MPa || 274.5 K	0.8996	5.1682	0.5121	0.0044	0.2752	1.2307	69.0062	0.4202

^{a} mol of guest gas^{1} mol of H_2O^{-1} m^{-2} min^{-1}; ^{b} J mol^{-1}.

Table S2. Optimal model parameters for CH_4 hydrate decomposition kinetics.

Case	Proposed model				Oyama et al.[39]
	α	K_0^{a} $\times 10^{-16}$	β_0	C	ϕ
CH_4 hydrate dissociation || Purewater || Silica sand || 75% water saturation || 4.8 MPa || 297.2 K	0.2800	1.0848	0.4339	0.0182	34.3968
CH_4 hydrate dissociation || 1.5 wt% salt solution || Silica sand || 75% water saturation || 4.8 MPa || 297.2 K	0.5640	1.0474	0.4713	0.0450	41.2815
CH_4 hydrate dissociation || 3.0 wt% salt solution || Silica sand || 75% water saturation || 4.8 MPa || 297.2 K	0.5031	1.0246	0.4099	0.0415	38.5033

^{a} mol of guest gas^{1} mol of H_2O^{-1} m^{-2} min^{-1}. Here, φ is the percentage contribution to the dissociation of the total heat input to the system.
